# Supplementary material for: Template-Free Preparation of α-Ni(OH)2 Nanosphere as High-Performance Electrode Material for Advanced Supercapacitor
Source: Nanomaterials (Basel). 2022 Jun 28;12(13):2216. doi: 10.3390/nano12132216 (PMC9267997; doi:10.3390/nano12132216)
Supplement: Supplementary file 1 [file nanomaterials-12-02216-s001.zip › nanomaterials-1766599-supplementary.pdf]

Supporting Information

Template-Free Preparation of α-$Ni(OH)_2$ Nanosphere as High-Performance Electrode Material for Advanced Supercapacitor

Rongrong Zhang [1,†], Qian Tu [1,†], Xianran Li [1], Xinyu Sun [1,*], Xinghai Liu [2,*] and Liangzhe Chen [1,*]

[1] School of Electronic Information Engineering, Jingchu University of Technology, Jingmen 448000, China; 200805001@jcut.edu.cn (R.Z.); tq010406@163.com (Q.T.); ggll925597@163.com (X.L.)

[2] Research Center of Graphic Communication, Printing and Packaging, Wuhan University, Wuhan 430079, China

* Correspondence: sxy007@jcut.edu.cn (X.S.); liuxh@whu.edu.cn (X.L); chen_lz1991@whu.edu.cn (L.C.)

† These authors contributed equally to this paper.

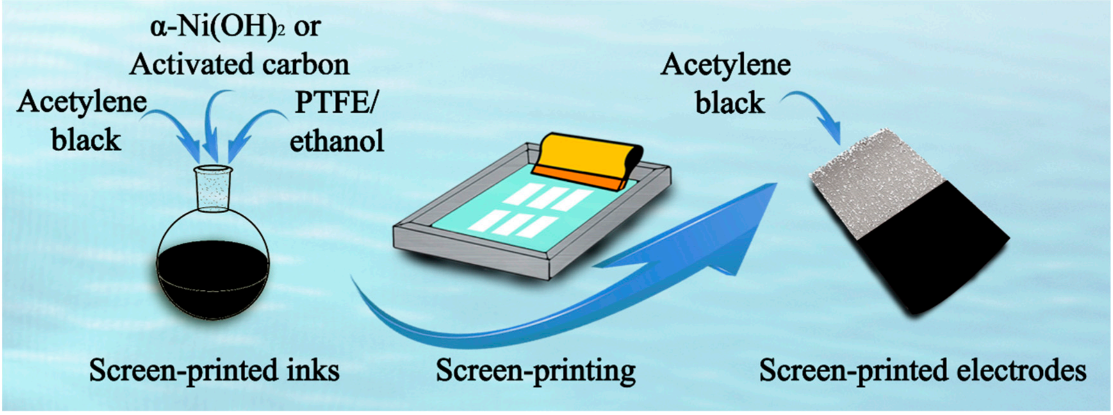

Figure S1. Diagram of the preparation of the screen-printed electrodes.

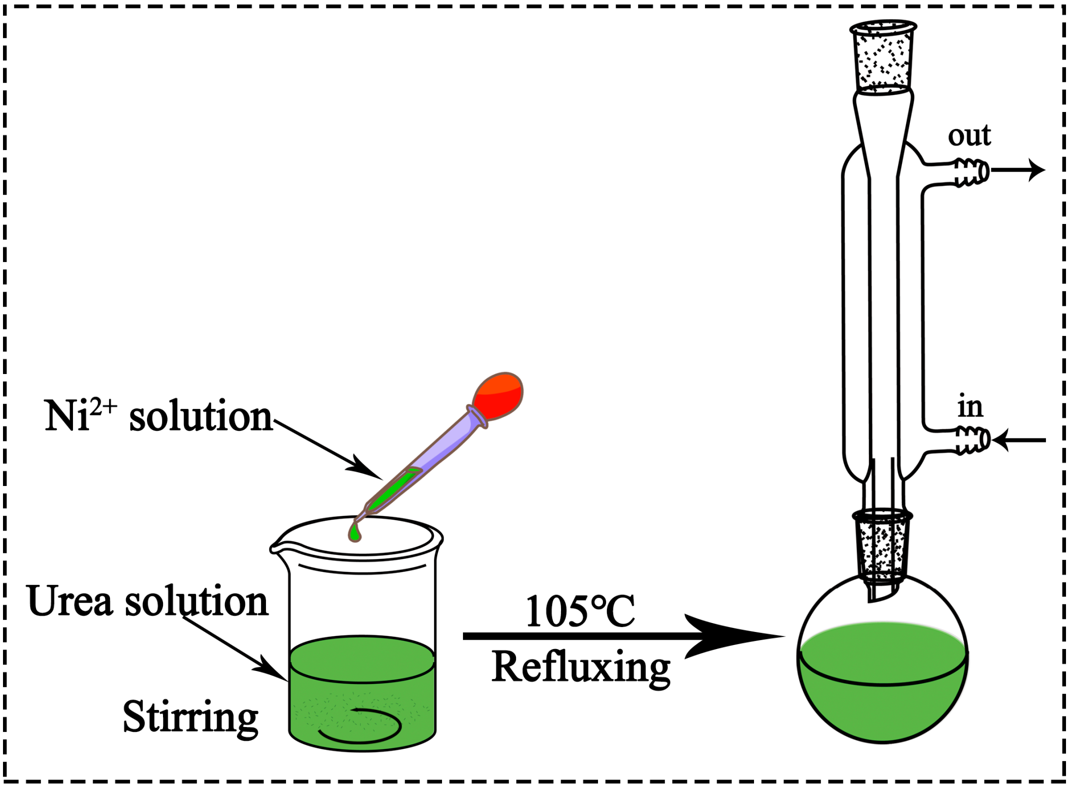

Figure S2. Schematic illustration for the preparation of the $Ni(OH)_2$ nanospheres.

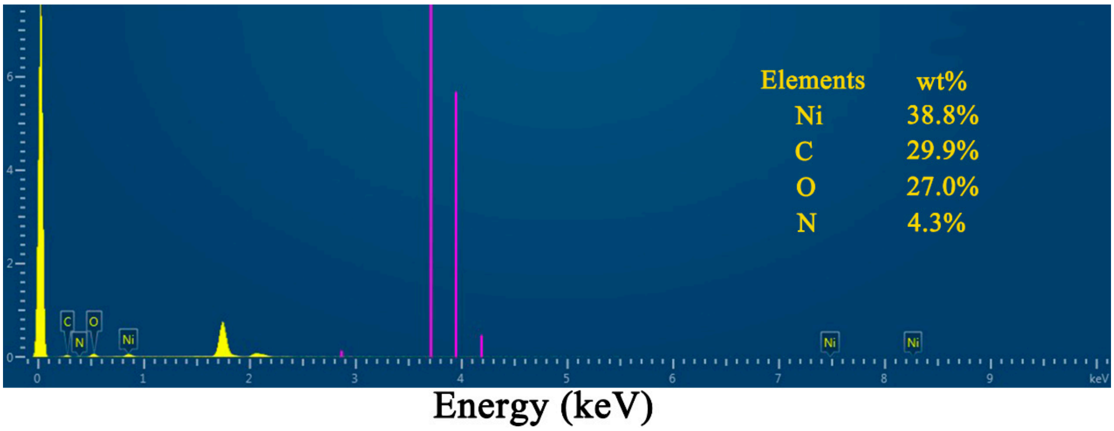

Figure S3. EDS spectrum of the $Ni(OH)_2$ nanospheres.

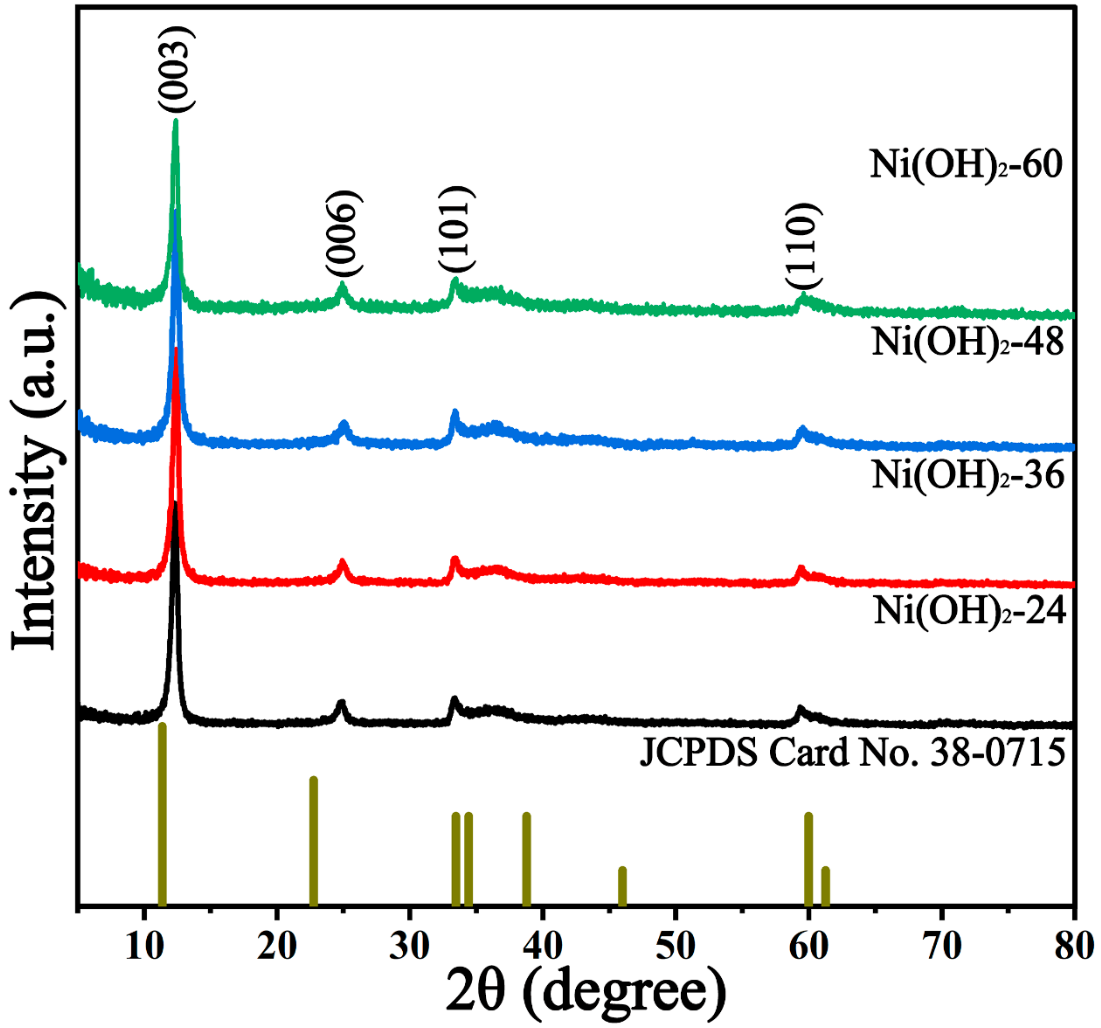

Figure S4. XRD patterns of $Ni(OH)_2$-24, $Ni(OH)_2$-36, $Ni(OH)_2$-48 and $Ni(OH)_2$-60.

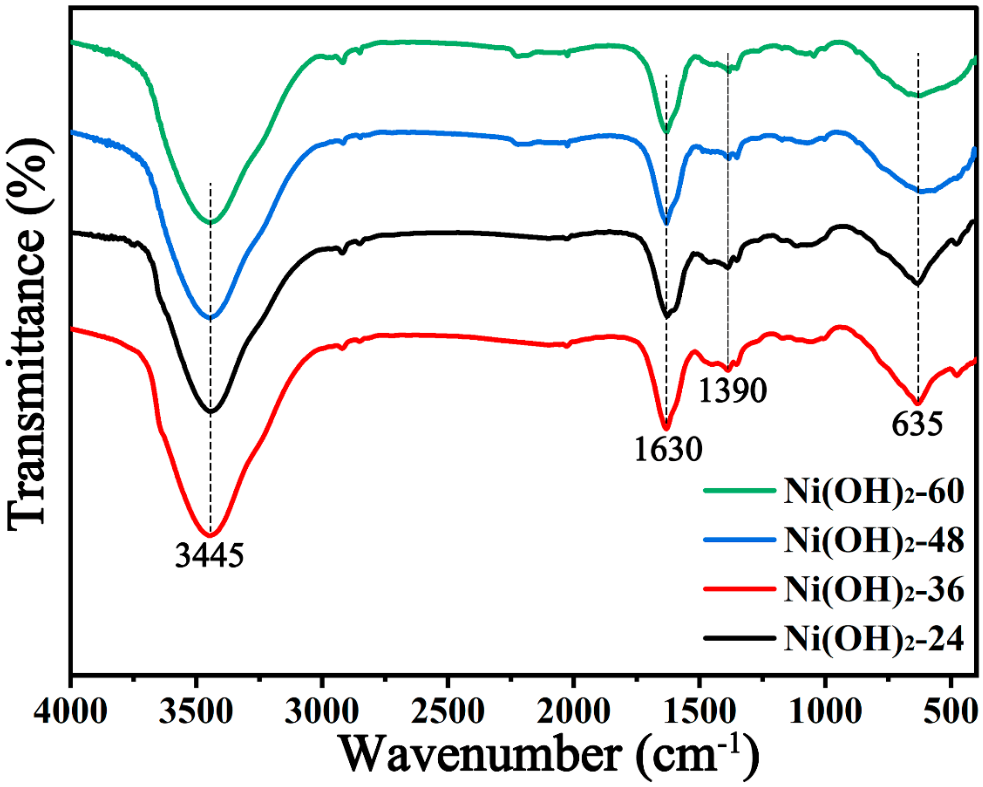

Figure S5. FT-IR spectra of $Ni(OH)_2$-24, $Ni(OH)_2$-36, $Ni(OH)_2$-48 and $Ni(OH)_2$-60.

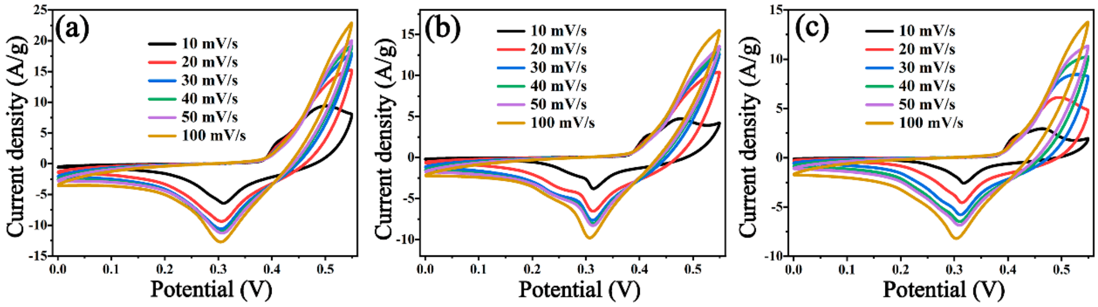

Figure S6. CV curves of (a) $Ni(OH)_2$-24, (b) $Ni(OH)_2$-48 and (c) $Ni(OH)_2$-60 electrodes.

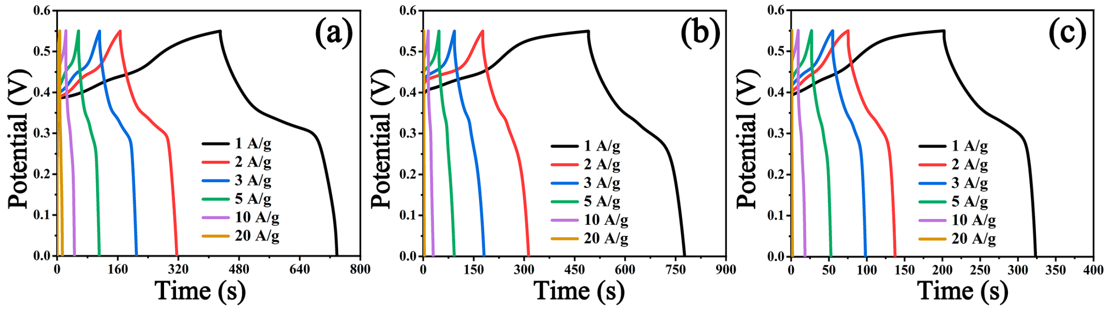

Figure S7. GCD curves of $Ni(OH)_2$-24, $Ni(OH)_2$-48 and $Ni(OH)_2$-60 electrodes.

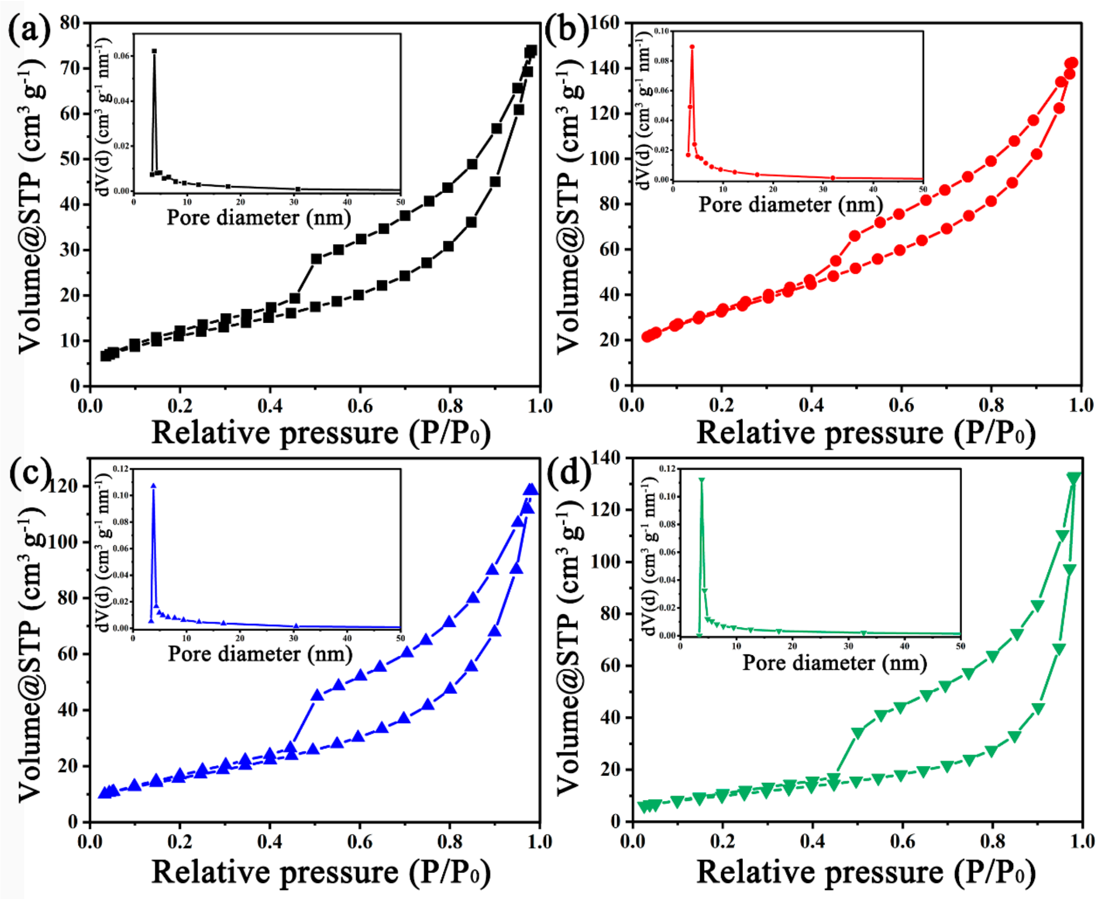

Figure S8. N_2 adsorption/desorption isotherm plots and the corresponding pore size distribution of (a) $Ni(OH)_2$-24, (b) $Ni(OH)_2$-36, (c) $Ni(OH)_2$-48 and (d) $Ni(OH)_2$-60.

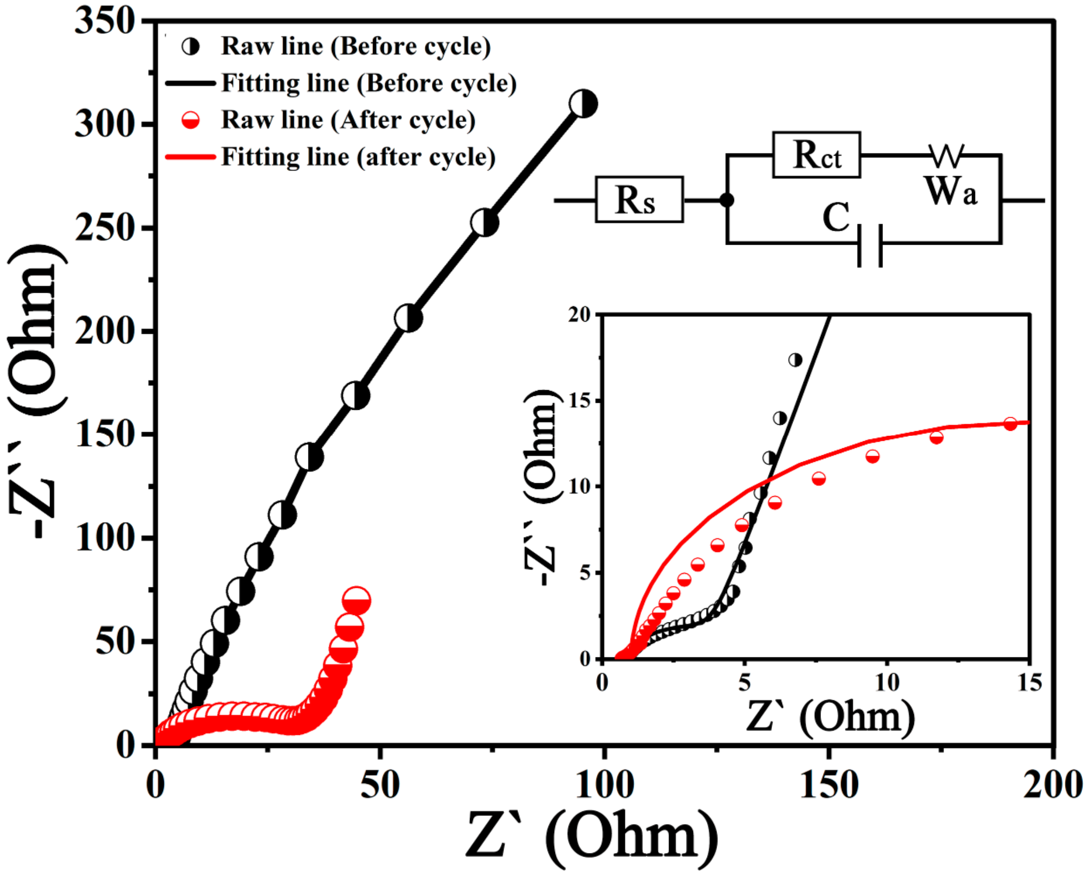

Figure S9. Nyquist plots of the $Ni(OH)_2$-36//AC ASC before and after cycles (the inset show the enlarge high-frequency region of the plot and the equivalent circuit).

Table S1. Specific capacitance comparison of $Ni(OH)_2$-based materials from literature reports.

Materials	Specific capacitance	Current density	Electrolyte	Refs.
$Ni(OH)_2$ nanoball	1243 F/g	1 A/g	6M KOH	This work
	1100 F/g	2 A/g		
	1025 F/g	3 A/g		
	951 F/g	5 A/g		
	828 F/g	10 A/g		
$Ni(OH)_2$ nanoplate	447 F/g	1 A/g	1M NaOH	[50]
$Ni(OH)_2$ sheet	693 F/g	4 A/g	6M KOH	[22]
$Ni(OH)_2$ thin film	980 F/g	10 mV/s	30wt% KOH	[51]
Ultrathin $Ni(OH)_2$	2064 F/g	2 A/g	6M KOH	[52]
$Ni(OH)_2$/GDL	418 F/g	1 A/g	1M NaOH	[53]
$Ni(OH)_2$-GS-CNT	1170 F/g	0.2 A/g	6M KOH	[54]
$Ni(OH)_2$@Mn_2O_3 flower	1219 F/g	2 A/g	6M KOH+ 0.3M LiOH	[55]
$Ni(OH)_2$-α-Fe_2O_3	1107 F/g	20 mV/s	1M KOH	[56]
$Ni(OH)_2$@xRF	672 F/g	2 A/g	2M KOH	[57]
$ZnCo_2S_4$@$Ni(OH)_2$	1193 C/g	1 A/g	3M KOH	[58]

Table S2. Specific surface area, total pore volume and average pore size parameters for $Ni(OH)_2$-24, $Ni(OH)_2$-36, $Ni(OH)_2$-48 and $Ni(OH)_2$-60.

Samples	Specific surface area (m^2g^{-1})[a]	Total pore volume (cm^3g^{-1})[b]	Average pore size (nm)[b]
$Ni(OH)_2$-24	42.0	0.108	3.807
$Ni(OH)_2$-36	119.4	0.190	3.401
$Ni(OH)_2$-48	59.2	0.175	3.794
$Ni(OH)_2$-60	37.1	0.202	3.793

[a] Obatined from BET method.

[b] Obatined from BJH method.

Table S3. The EIS parameters of *Rs*, *Rct*, *Wa* and *C* for $Ni(OH)_2$-24, $Ni(OH)_2$-36, $Ni(OH)_2$-48 and $Ni(OH)_2$-60 electrodes.

Parameters	Rs/Ω	Rct/Ω	Wa/Ω	C/F
$Ni(OH)_2$-24	0.87	0.48	464.59	0.00017
$Ni(OH)_2$-36	0.76	0.35	995.62	0.00032
$Ni(OH)_2$-48	0.78	0.89	926.66	0.00014
$Ni(OH)_2$-60	0.74	0.99	733.26	0.00013

Table S4. The EIS parameters of *Rs*, *Rct*, *Wa* and *C* for the $Ni(OH)_2$-36//AC ASC before and after cycles.

Parameters	Rs/Ω	Rct/Ω	Wa/Ω	C/F
Before	0.99	3.38	1968.90	0.000059
After	1.01	27.08	418.58	0.0032
